# Dataset on human rights awareness in Northwest Nigeria

**DOI:** 10.1016/j.dib.2021.107547

**Published:** 2021-11-06

**Authors:** Kasim Balarabe

**Affiliations:** O. P. Jindal Global University, Sonipat, India

**Keywords:** Human rights, Human rights claim, Human rights awareness, Human rights complaints, Comfortability in litigating, Northwest Nigeria

## Abstract

The data in this article investigated the extent of human rights awareness in the seven States comprising the Northwest geopolitical zone of Nigeria and its relationship with the characteristics of the population in the light of limited human rights claims in the region. The data was obtained from 780 respondents using stratified and systematic random sampling techniques using with the help of a structured questionnaire. It is aimed at ascertaining, among others, the extent to which the population in the region is aware of human rights and the meaning of the terms ‘marginalisation' and ‘discrimination', whether the population is aware of how to claim human rights and whether the population is comfortable approaching the courts for human rights claims. The questionnaire also sought information on the most utilised sources of information, formal and informal factors that influence a decision to litigate human rights violations, and the most common complaint mechanisms employed by the population concerning human rights violations. The responses were analysed using Qualtrics software, and the data was presented using statistical representations. The data shows an appreciable level of human rights understanding in the region.

## Specifications Table

**Table:** 

Subject	Social Science
Specific subject area	Law
Type of data	Map Table
How data were acquired	For data collection in this article, a 33-questions structured questionnaire was administered face to face. The data was collected between 17 November 2020 – 13 January 2021. The statistical population consisted of 780 people from the seven States comprising the Northwest geopolitical zone of Nigeria.
Data format	Raw Analysed
Parameters for data collection	The survey addressed randomly selected Nigerian citizens older than 18 residing in the State capitals and the randomly selected local governments.
Description of data collection	Face-to-face survey.
Data source location	Seven States comprise the Northwest political zone of Nigeria. These are: Dutse and Babura of Jigawa State, Nigeria. Kaduna and Giwa of Kaduna State, Nigeria. Kano and Bunkure of Kano State, Nigeria. Katsina and Dutsin-ma of Katsina State, Nigeria. Birnin-Kebbi and Maiyama of Kebbi State, Nigeria Sokoto and Yabo of Sokoto State, Nigeria, Gusau and Gummi of Zamfara State, Nigeria
Data accessibility	Data is accessible via Mendeley Data: Balarabe, Kasim (2021), ``Dataset on Human Rights Awareness and the Right to Water and Sanitation in Northwest Nigeria”, Mendeley Data, http://doi.org/10.17632/x46s9zj8n6.1[4]

## Value of the Data

• The data presents the extent of human rights awareness and how to claim human rights in the event of violations. The data also shows how the population feels comfortable approaching the courts to assert legal claims for human rights, including factors that influence their decision to claim human rights through the courts. Thus, the data is valuable in understanding the characteristics of the population, the opportunities that can be utilised to enable the people to promote and protect their human rights and the human rights challenges in the region.
• The data is useful to research institutions, international and national human rights organisations and institutions, civil society organisations, human rights defenders, and activists.
• The data can be used to ascertain the appropriate intervention needed to design programmes and projects that can empower the population in realising human rights in the selected States.

## 1 Data Description

The generated data from the survey is deposited on the Mendeley data website. It is raw data that contains, among others, responses on the participants’ human rights awareness and the right to water and sanitation in the seven States comprising Northwest Nigeria. The data include the demographic information of the participants, their sources of information, responses to questions on human rights awareness, comfortability, and willingness to litigate human rights violations, availability of and accessibility to water sources, and factors that impact their decision and willingness to assert human rights claims.

In this article, the data comprise one map and eight tables. The map indicates the Northwest geopolitical zone of Nigeria, which contains seven States from where the author collected the 780 responses. It is the region with the highest population among the six zones in Nigeria. Arguably, the region has the highest number of human rights violations [1]. Nigeria continues to experience human rights challenges politically, economically, and socially and perpetrators are not often held accountable [2]. Not only that there is a culture of impunity, but the population is also docile in terms of vigorously asserting human rights claims against the perpetrators. It is crucial to investigate whether the population is aware of its rights and how to claim them.

Table 1 shows the demographics of survey respondents tabulated into gender, age group, marital status, religion, education, and occupation. The highest percentage of the respondents is male (67.69%), and respondents from 25-40 years constitute the highest percentage (48.33%). The data in Table 1 also shows a slight variation between married and single respondents (0.12%), and in the context of religion, Muslims constituted 84.36% of the total number of respondents. Educationally, possessors of secondary school certificates formed the highest number of respondents, followed by national diploma holders. Students have the highest number (32.56%) in terms of occupation, followed by civil servants with 27.18%.

**Figure:**
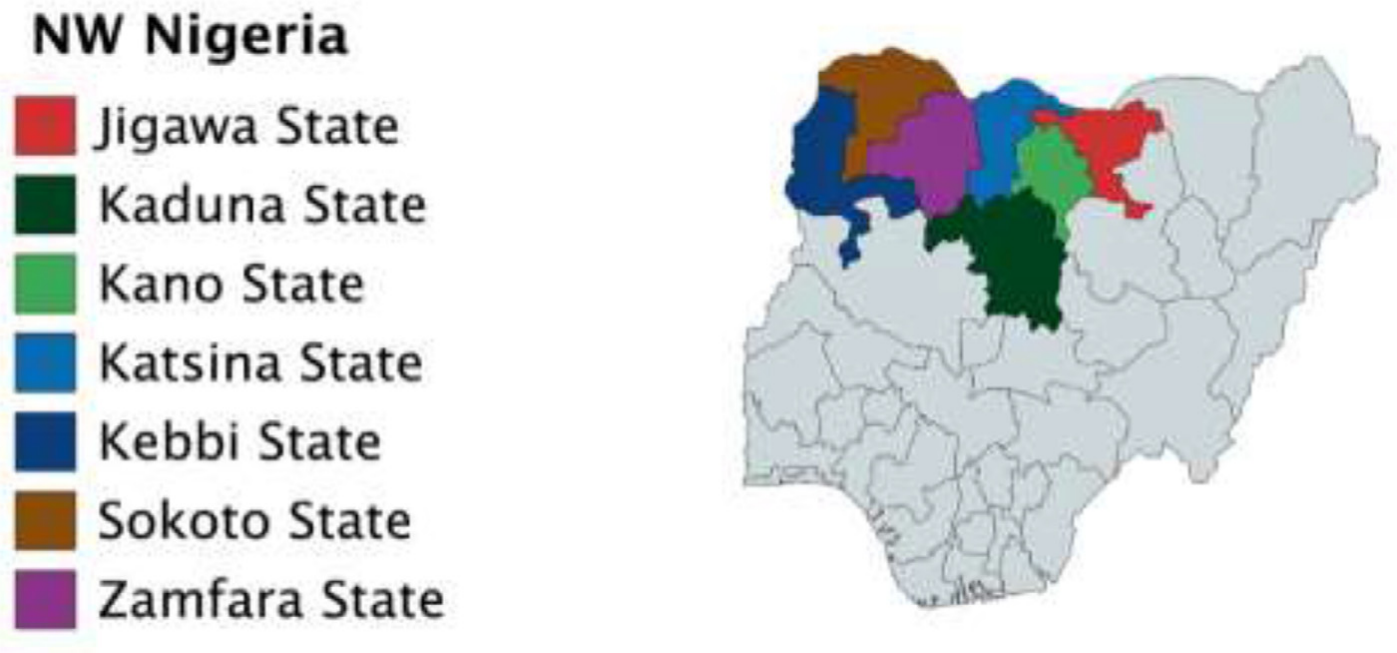

**Table 1:** Demographics of Survey Respondents.

Variable	N (%)	Total
**Gender**		
*Male*	528 (67.69%)	
*Female*	252 (32.31%)	
		**780**
**Age Group**		
*18-24*	249 (31.92%)	
*25-40*	377 (48.33%)	
*41-50*	122 (15.64%)	
*50+*	32 (4.10%)	
		**780**
**Marital Status**		
*Single*	376 (48.21%)	
*Married*	377 (48.33%)	
*Widow*	9 (1.15%)	
*Divorced*	8 (1.03%)	
*Other*	10 (1.28%)	
		**780**
**Religion**		
*Islam*	658 (84.36%)	
*Christianity*	122 (15.64%)	
		**780**
**Education**		
*Primary*	54 (6.92%)	
*Secondary*	249 (31.92)	
*Diploma*	223 (28.59)	
*Bachelor*	146 (18.72%)	
*PGD*	22 (2.82%)	
*Masters*	15 (1.92%)	
*PhD*	2 (0.26%)	
*Others*	69 (8.85%)	
		**780**
**Occupation**		
*Civil Servants*	212 (27.18%)	
*Private Sector*	75 (9.62%)	
*Farming*	35 (4.49%)	
*Self-employed*	91 (11.67%)	
*Housewives*	44 (5.64%)	
*Retired*	3 (0.38%)	
*Students*	254 (32.56%)	
*Unemployed*	47 (6.03%)	
*Others*	19 (2.44%)	
		**780**

Table 2 shows respondents' most utilised sources of information on human rights issues. The data were tabulated into six different utilisation levels, from the minor to the highest and the total number of respondents who answered the question. The data shows social media (Facebook, Twitter, Instagram etc.) is the most frequently utilised source, and the least used are books.

**Table 2:** Respondents’ Sources of Information on Human Rights and Frequency of Utilisation in Northwest Nigeria.

Variable	Never	Almost never	Sometimes	Fairly often	Very often	Always	Total
**Radio**	132 (16.92%)	54 (6.92%)	191 (24.49%)	84 (10.77%)	92 (11.78%)	227 (29.10%)	**780**
**Television**	65 (8.33%)	51 (6.54)	176 (22.56%)	87 (11.15%)	125 (16.03%)	237 (30.38%)	**741**
**Newspaper**	224 (28.72%)	64 (8.21%)	181 (23.21%)	70 (8.97%)	81 (10.38%)	118 (15.13%)	**738**
**Books**	288 (36.92%)	77 (9.87%)	90 (11.54%)	57 (7.31%)	77 (9.87%)	118 (15.13%)	**707**
**Internet**	160 (20.51%)	40 (5.13%)	140 (17.95%)	95 (12.18%)	95 (12.18%)	223 (28.59%)	**753**
**Social media**	128 (16.41%)	34 (4.36%)	126 (16.15%)	56 (7.18%)	131 (16.79%)	272 (34.87%)	**747**

**Table 3:** Human Rights Awareness in Northwest Nigeria.

Variable	Yes	No	Do not care	Total
**General**	681 (87.87%)	72 (9.29%)	22 (2.84%)	**775**
**Gender**				
*Male*	460 (87.79%)	45 (8.59%)	19 (3.63%)	524
*Female*	221 (88.05%)	27 (10.76%)	3 (1.20%)	251
				**775**
**Age Group**				
*18-24*	219 (88.66%)	25 (10.12%)	3 (1.21%)	247
*25-40*	331 (88.27%)	31 (8.27%)	13 (3.47%)	375
*41-50*	110 (90.91%)	8 (6.61%)	3 (2.48%)	121
*50+*	21 (65.63%)	8 (25.00%)	3 (9.38%)	32
				**775**
**Education**				
*Primary*	38 (71.70%)	10 (18.87%)	5 (9.43%)	53
*Secondary*	211 (85.08%)	33 (13.31%)	4 (1.61%)	248
*Diploma*	211 (95.05%)	9 (4.05%)	2 (0.90%)	222
*Bachelor*	130 (90.28.04%)	9 (6.25%)	5 (3.47%)	144
*PGD*	22 (100.00%)	0 (0.00%)	0 (0.00%)	22
*Masters*	15 (100.00%)	0 (0.00%)	0 (0.00%)	15
*PhD*	2 (100.00%)	0 (0.00%)	0 (0.00%)	2
*Others*	52 (75.36%)	11 (15.94%)	6 (8.70%)	69
				**775**
**Occupation**				
*Civil Servants*	191 (91.39%)	14 (6.70%)	4 (1.91%)	209
*Private Sector*	69 (92.00%)	2 (2.67%)	4 (5.33%)	75
*Farming*	23 (65.71%)	8 (22.86%)	4 (11.43%)	35
*Self-employed*	74 (81.32%)	13 (14.29%)	4 (4.40%)	91
*Housewives*	39 (88.64%)	5 (11.36%)	0 (0.00%)	44
*Retired*	3 (100.00%)	0 (0.00%)	0 (0.00%)	3
*Students*	227 (90.08%)	22 (8.73%)	3 (1.90%)	252
*Unemployed*	37 (78.72%)	7 (14.89%)	3 (6.38%)	47
*Others*	18 (94.74%)	1 (5.26%)	0 (0.00%)	19
				**775**

Table 3 highlights the extent of human rights awareness in the region. It indicates that most respondents (87.87%) are aware of what human rights entail, 9.29% said they are not, and 2.84% showed they do not care. The data was further cross-tabulated into gender, age group, education, and occupation. In terms of gender, there is no significant variation in percentage between males and females. By age group, those aged 41-50 years account for the highest number of those who claim to be aware (90.91%). The table also shows the percentages in terms of education and occupation. Similar information is shown by Table 4 on awareness of the meaning of marginalisation and discrimination, which appeared to be lower compared to awareness of human rights.

**Table 4:** Awareness of the Term `Marginalisation' or `Discrimination'.

Variable	Yes	No	Total
**General**	604 (79.06%)	160 (20.94%)	**764**
**Gender**			
*Male*	413 (79.73%)	105 (20.27%)	518
*Female*	191 (77.64%)	55 (22.36%)	246
			**764**
**Age Group**			
*18-24*	174 (71.60%)	69 (28.40%)	243
*25-40*	302 (81.84%)	67 (18.16%)	369
*41-50*	107 (88.43%)	14 (11.57%)	121
*50+*	21 (67.74%)	10 (32.26%)	31
			**764**
**Education**			
*Primary*	37 (71.15%)	15 (28.85%)	52
*Secondary*	190 (79.17%)	50 (20.83%)	240
*Diploma*	166 (74.44%)	57 (25.56%)	223
*Bachelor*	121 (85.21%)	21 (14.79%)	142
*PGD*	20 (90.91%)	5 (9.09%)	22
*Masters*	14 (100.00%)	0 (0.00%)	14
*PhD*	2 (100.00%)	0 (0.00%)	2
*Others*	54 (78.26%)	15 (21.74%)	69
			**764**
**Occupation**			
*Civil Servants*	175 (83.33%)	35 (16.67%)	210
*Private Sector*	61 (82.43%)	13 (17.57%)	74
*Farmers*	24 (68.57%)	11 (31.43%)	35
*Self-Employed*	70 (79.55%)	18 (20.45%)	88
*Housewives*	33 (76.74%)	10 (23.26%)	43
*Retired*	3 (100.00%)	0 (0.00%)	3
*Students*	189 (76.52%)	58 (23.48%)	247
*Unemployed*	33 (73.33%)	12 (26.67%)	45
*Others*	16 (84.21%)	3 (15.79%)	19
			**764**

Table 5 shows data concerning awareness of how to claim human rights where there is a violation. In general, 72.98% indicated they are aware of how to claim their rights, and 20.36% indicated they are not, while 6.66% do not care. In Table 6, respondents reveal whether they are comfortable litigating human rights violations. From the data, 62.12% of the respondents feel comfortable accessing courts for human rights claims and 37.88% said they are uncomfortable. When the data were cross-tabulated, the responses demonstrated that in terms of gender, there is a slight variation between males and females of about 9% in favour of males. In terms of age, the data showed only slight variation between the groups. In terms of marital status, the difference between the highest (widow group) and the lowest (divorced group) is about 16% in favour of widows. In the context of religion, the difference is between the margin of error. The table also shows different levels of responses when the data were cross-tabulated by educational qualifications and occupation.

**Table 5:** Awareness of How to Claim Human Rights.

Variable	Yes	No	Do not care	Total
**General**	559 (72.98%)	156 (20.36%)	51 (6.66%)	**766**
**Gender**				
*Male*	383 (73.65%)	101 (19.42%)	36 (6.92%)	520
*Female*	176 (71.54%)	55 (22.36%)	15 (6.10)	246
				**766**
**Age Group**				
*18-24*	177 (72.54%)	53 (21.72%)	14 (5.74%)	244
*25-40*	265 (72.01%)	76 (20.65%)	27 (7.34%)	368
*41-50*	96 (78.69%)	20 (16.39%)	6 (4.92%)	122
*50+*	21 (65.63%)	7 (21.88%)	4 (12.50%)	32
				**766**
**Education**				
*Primary*	31 (59.61%)	15 (23.08%)	6 (11.54%)	52
*Secondary*	171 (69.51%)	57 (23.17%)	18 (7.32%)	246
*Diploma*	172 (78.18%)	36 (16.36%)	12 (5.45%)	220
*Bachelor*	102 (72.34%)	28 (19.86%)	11 (7.80%)	141
*PGD*	20 (90.91%)	2 (9.09%)	0 (0.00%)	22
*Masters*	14 (93.33%)	1 (6.67%)	0 (0.00%)	15
*PhD*	2 (100.00%)	0 (0.00%)	0 (0.00%)	2
*Others*	47 (69.12%)	17 (25.00%)	4 (5.88%)	68
				**766**
**Occupation**				
*Civil Servants*	160 (78.05%)	34 (16.59%)	11 (5.37%)	205
*Private Sector*	51 (68.00%)	15 (20.00%)	9 (12.00%)	75
*Farmers*	15 (42.86%)	10 (28.57%)	10 (28.57%)	35
*Self-Employed*	62 (68.13%)	21 (23.08%)	8 (8.79%)	91
*Housewives*	31 (72.09%)	11 (25.58%)	1 (2.33%)	43
*Retired*	3 (100.00%)	0 (0.00%)	0 (0.00%)	3
*Students*	191 (76.71%)	47 (18.88%)	11 (4.42%)	249
*Unemployed*	31 (67.39%)	14 (30.43%)	1 (2.17%)	46
*Others*	15 (78.95%)	4 (21.05%)	0 (0.00%)	19
				**766**

**Table 6:** Comfortability Litigating for Human Rights Violations.

Variable	Yes	No	Total
**General**	474 (62.12%)	289 (37.88%)	**763**
**Gender**			
Male	337 (65.06%)	181 (34.94%)	518
Female	137 (55.92%)	108 (43.08%)	245
			**763**
**Age Group**			
18-24	146 (60.83%)	94 (39.17%)	240
25-40	231 (62.26%)	140 (37.74%)	371
41-50	79 (65.83%)	41 (34.17%)	120
51+	18 (56.25%)	14 (43.75%)	32
			**763**
**Marital Status**			
Single	219 (60.16%)	145 (39.84%)	364
Married	239 (64.25%)	133 (35.75%)	372
Widow/Widower	6 (66.67%)	3 (33.33%)	9
Divorced	4 (50.00%)	4 (50.00%)	8
Others	6 (60.00%)	4 (40.00%)	10
			**763**
**Religious Belief**			
Islam	403 (62.58%)	241 (37.42%)	644
Christianity	71 (59.66%)	48 (40.34%)	119
			**763**
**Education**			
Primary	28 (52.83%)	25 (47.17%)	53
Secondary	140 (57.85%)	102 (42.15%)	242
Diploma	152 (69.09%)	68 (30.91%)	220
Bachelor	92 (62.25%)	49 (34.75%)	141
PGD	17 (80.95%)	4 (19.05%)	21
Masters	12 (80.00%)	3 (20.00%)	15
PhD	1 (50.00%)	1 (50.00%)	2
Other	32 (46.38%)	37 (53.62%)	69
			**763**
**Occupation**			
Civil Servants	147 (71.14%)	59 (28.86%)	206
Private Sector	49 (66.22%)	26 (33.78%)	75
Farming	16 (40.00%)	18 (60.00%)	34
Self-employed	49 (55.68%)	39 (44.32%)	88
Housewives	23 (53.49%)	20 (46.51%)	43
Retired	2 (66.67%)	1 (33.33%)	3
Students	151 (60.48%)	98 (39.52%)	249
Unemployed	26 (57.17%)	20 (42.86%)	46
Others	11 (57.89%)	8 (42.11%)	19
			**763**

Table 7 illustrates factors that influence a decision to litigate human rights violations. The most common factors in the region are culture and tradition, religious belief, family influence, the financial situation of the litigant, traditional ruler, government bureaucracy and distance between the litigant and the courts. The table shows the extent of the impact each of these factors has on the decision to litigate. Lastly, Table 8 shows some of the most utilised complaints mechanisms for human rights violations. Respondents indicated the extent to which they complain to traditional rulers, courts, human rights institutions, civil society organisations, the media, and the police.

**Table 7:** Factors Impacting on Human Rights Claims in Northwest Nigeria.

Variable	Very Unlikely	Unlikely	Somewhat Unlikely	Neutral	Somewhat Likely	Likely	Very Likely	Total
Culture and Tradition	316 (43.65%)	79 (10.91%)	28 (3.87%)	155 (21.41%)	28 (3.87%)	35 (4.83%)	83 (11.46%)	**724**
Religious Belief	274 (37.95%)	69 (9.56%)	21 (2.91%)	134 (18.56%)	33 (4.57%)	61 (8.45%)	130 (18.00%)	**722**
Family Influence	251 (35.10%)	68 (9.51%)	32 (4.48%)	139 (19.44%)	48 (6.71%)	72 (10.07%)	105 (14.69)	**715**
Financial Situation	194 (27.48%)	43 (6.09%)	34 (4.82%)	138 (19.55%)	47 (6.67%)	75 (10.62%)	175 (24.79%)	**706**
Traditional Ruler	255 (36.32%)	72 (10.26%)	46 (6.55%)	184 (26.21%)	40 (5.70%)	49 (6.98%)	56 (7.98%)	**702**
Bureaucracy	228 (31.93%)	60 (8.40%)	36 (5.04%)	186 (26.05%)	48 (6.72%)	71 (9.94%)	85 (11.90%)	**714**
Distance	235 (34.00%)	56 (8.10%)	38 (5.50%)	184 (26.63%)	49 (7.09%)	55 (7.96%)	74 (10.71%)	**691**

**Table 8:** Common Complaints Mechanisms for Human Rights Violation in Northwest Nigeria.

Variable	Never	Almost never	Sometimes	Fairly often	Very often	Always	Total
Traditional rulers	378 (51.71%)	59 (8.07%)	120 (16.42%)	50 (6.84%)	62 (8.48%)	62 (8.48%)	**731**
Courts	520 (72.42%)	70 (9.75%)	51 (7.10%)	28 (3.90%)	19 (2.65%)	30 (4.18%)	**718**
HR institutions	423 (59.83%)	85 (12.02%)	91 (12.87%)	34 (4.81%)	32 (4.53%)	42 (5.94%)	**707**
Civil society	353 (50.72%)	75 (10.78%)	113 (16.24%)	56 (8.05%)	52 (7.47%)	47 (6.75%)	**696**
Media	317 (44.96%)	74 (10.50%)	105 (14.89%)	63 (8.94%)	66 (9.36%)	80 (11.35%)	**705**
Police	396 (55.31%)	60 (8.38%)	102 (14.25%)	43 (6.00%)	35 (4.89%)	80 (11.17%)	**716**

## 2 Experimental Design, Materials and Methods

The researcher conducted the empirical study in the seven States comprising the Northwest geopolitical zone of Nigeria. These States are Jigawa, Kaduna, Kano, Katsina, Kebbi, Sokoto and Zamfara. The author collected 780 responses based on the population to give a 95% confidence level and a 3.51% margin of error. The researcher took several steps to collect and analyse the data. The first step was selecting and training two assistants (male and female) who speak English and the local language to administer the questionnaires. In the second step, the researcher automatically included the State capitals to represent the urban areas of the States. In the third step, one local government was randomly selected from each of the seven States using Excel Software. In the fourth step, the researcher applied a stratified random sampling method to divide the selected cities and local governments into clusters. In the fifth step, using a systematic random sampling method, the researcher selected the households from each cluster where the questionnaires will be administered. In the final stage, the researcher and the assistants visited the houses of the selected participants and administered the questionnaires in English and Hausa languages to only the adult members of the household who are 18 years and above. The female assistants administered the questionnaires to housewives (who could not interact with males outside). Where necessary, a follow-up interview was conducted for clarifications concerning the open-ended questions.

The questionnaire contained an informed consent clause which each respondent read and signed before completing it. Except in a few instances, the questions were in the closed format. The researcher used leading questions, importance questions, Likert questions, dichotomous questions, bipolar questions, and rating scale questions. These provided the opportunity for a standardised way of measuring responses [3]. In cases where the opinion of the relevant stakeholder is relevant, the questionnaire contains open-ended questions. All the responses generated were entered in Qualtrics software and analysed using statistical representations (number of responses, percentages etc.) to provide insights into human rights in the region.

## Ethics Statement

There is no requirement for ethical approval to conduct this type of survey in Northwest Nigeria. However, participation in this study was entirely voluntary. All the participants read and signed an informed consent statement attached to the questionnaire before responding.

## CRediT Author Statement

**Kasim Balarabe**: Conceptualisation, Investigation, Data curation, Writing, Editing and Reviewing the manuscript.

## Data Availability

Dataset on Human Rights Awareness and the Right to Water and Sanitation in Northwest Nigeria (Original data) (Mendeley Data).

## Declaration of Competing Interest

The author declares that there is no known competing financial interests or personal relationships which have or could be perceived to have influenced the work reported in this article.

## Appendix Supplementary materials

Image, application 1
