# Supplementary material for: Dataset on human rights awareness in Northwest Nigeria
Source: Data Brief. 2021 Nov 6;39:107547. doi: 10.1016/j.dib.2021.107547 (PMC8601984; doi:10.1016/j.dib.2021.107547)
Supplement: Supplementary file 1 [file mmc1.docx]

Dear Respondent,

This survey is part of a research that seeks to identify the factors militating against the realisation of human rights and access to quality water and basic sanitation in Northwest Nigeria and to recommend best possible options to address the situation.

Your participation in this study is voluntary and your opinion is considered vital and extremely important. You are kindly requested to fill the attached questionnaire honestly and to the best of your ability. Your data will be treated as confidential, and all responses are anonymous and would not in any way be used to identify you as a person. Please sign below to indicate your consent to give your opinion on these issues.

Thank you.

____________________________

Signature and Date

SECTION ONE

1. Gender Male Female
2. Age group 18-24 25-40 41-50 51& above I don’t know Refused
3. Marital status Single Married Widow/Widower Divorced Other
4. Religion Islam Christianity Traditional Refused
5. Highest qualification Primary Secondary Diploma Bachelor PGD Masters PhD Other
6. Current occupation Civil Servant Private Sector Farming Self-employed Housewife Retired Student Unemployed Other

SECTION TWO:

1. How often do you use any of the following as a source of information? (Circle each one appropriately)

|  | Never | Almost never | Sometimes | Fairly often | Very often | Always |
| --- | --- | --- | --- | --- | --- | --- |
| Radio |  |  |  |  |  |  |
| Television |  |  |  |  |  |  |
| Newspaper/Magazine |  |  |  |  |  |  |
| Internet |  |  |  |  |  |  |
| Books |  |  |  |  |  |  |
| Social media (Facebook, Twitter etc) |  |  |  |  |  |  |
| Other |  |  |  |  |  |  |

1. Are you aware of the meaning of human rights? Yes No I don’t care
2. If any of your economic, social or cultural right (e.g. right to education, health, housing, development, clean environment) is denied, are you aware of how to claim it?

| Yes | No | I don’t care |
| --- | --- | --- |

1. Do you feel comfortable going to court to claim your rights? Yes No
2. If you are to know that the following are your human rights but are being denied what is the likelihood that you will claim them? (Please circle each one appropriately)

|  | Very unlikely | Unlikely | Somewhat unlikely | Neutral | Somewhat likely | Likely | Very likely |
| --- | --- | --- | --- | --- | --- | --- | --- |
| Right to food |  |  |  |  |  |  |  |
| Right to housing |  |  |  |  |  |  |  |
| Right to water |  |  |  |  |  |  |  |
| Right to clean environment |  |  |  |  |  |  |  |
| Right to work |  |  |  |  |  |  |  |
| Right to health |  |  |  |  |  |  |  |

1. If you have ever channelled a complaint or grievance, please circle the rate at which you use any of the following: (Please circle each one appropriately)

|  | Never | Almost never | Sometimes | Fairly often | Very often | Always |
| --- | --- | --- | --- | --- | --- | --- |
| Traditional Rulers |  |  |  |  |  |  |
| Courts |  |  |  |  |  |  |
| Human Rights Institution |  |  |  |  |  |  |
| Civil Society/NGOs |  |  |  |  |  |  |
| Media |  |  |  |  |  |  |
| Police |  |  |  |  |  |  |

SECTION THREE

1. Please rate the source from which you mostly get your drinking water (please circle each one appropriately)

|  | Never | Almost never | Sometimes | Fairly often | Very often | Always |
| --- | --- | --- | --- | --- | --- | --- |
| Personal Pump |  |  |  |  |  |  |
| Public Pump |  |  |  |  |  |  |
| Public Borehole |  |  |  |  |  |  |
| Personal Borehole |  |  |  |  |  |  |
| Vendor |  |  |  |  |  |  |
| River |  |  |  |  |  |  |
| Pond |  |  |  |  |  |  |
| Well |  |  |  |  |  |  |
| Other |  |  |  |  |  |  |

1. If your water source is not at home, what is the approximate distance from your house?

Less than one kilometre One kilometre More than one kilometre

1. If your source of water is personal borehole or personal well, is there a need to seek the approval of government before construction? Yes No
2. Is your water clean? Yes No To some extent
3. On the average, how much do you spend on water daily? 0 Naira 1-100 Naira 101-200 Naira 201-300 Naira More than 300 Naira
4. How long does it take you to fetch water from your house to the water source?

0-15 Minutes 15-30 Minutes 30-60 Minutes More than 60 Minutes

1. In your opinion, who do think is responsible for providing you with water?

Government Private Sector Vendors Myself

1. In your opinion do you think access to water and sanitation is your human right entitlement? Yes No I don’t know
2. Do you know where you can get information about water and sanitation?

Yes No I don’t care

1. Have you ever approached any office or agency of the government regarding the issue of water or sanitation? Yes No I don’t care
2. Have you already heard about the right to water and sanitation? Yes No
3. In the last two years, which of the following have you approached regarding provision of access to water and sanitation? Politician Traditional Ruler Court Civil Society None
4. Please rate how any of the following can prevents you from accessing the court to assert a claim to the right to water and sanitation (please circle)

|  | Very unlikely | Unlikely | Somewhat unlikely | Neutral | Somewhat likely | Likely | Very likely |
| --- | --- | --- | --- | --- | --- | --- | --- |
| Culture/tradition |  |  |  |  |  |  |  |
| Religious belief |  |  |  |  |  |  |  |
| Family |  |  |  |  |  |  |  |
| Money |  |  |  |  |  |  |  |
| Traditional Ruler |  |  |  |  |  |  |  |
| Gov’t bureaucracy |  |  |  |  |  |  |  |
| Distance |  |  |  |  |  |  |  |

SECTION FOUR

1. Are you aware of the term “marginalisation” or “discrimination”? Yes No
2. Do you have any marginalised/discriminated group in your area?

Yes No I don’t know

1. Are you aware of any group of people that has never been covered by water development projects in Nigeria? Yes No I don’t know
2. If yes, rate the extent to which in your opinion is the reason for their non-inclusion. (Please circle each one appropriately)

|  | Strongly agree | Agree | Neither agree nor disagree | Disagree | Strongly disagree |
| --- | --- | --- | --- | --- | --- |
| Distance of the area |  |  |  |  |  |
| Inaccessibility |  |  |  |  |  |
| Population is small |  |  |  |  |  |
| Status of the people (minority, language etc.) |  |  |  |  |  |
| They are poor) |  |  |  |  |  |
| Disability |  |  |  |  |  |
| Gender |  |  |  |  |  |
| Other |  |  |  |  |  |

1. When planning, establishing or constructing water and sanitation projects in your area, have you ever been part of or been consulted by the Government? Yes No

SECTION FIVE

1. To what extend do you think the following can influence access to water and sanitation in your area? (Please circle each one appropriately)

|  | Very unlikely | Unlikely | Somewhat unlikely | Neither likely nor unlikely | Somewhat likely | Likely | Very likely |
| --- | --- | --- | --- | --- | --- | --- | --- |
| Traditional Rulers |  |  |  |  |  |  |  |
| Civil Society/ NGOs |  |  |  |  |  |  |  |
| Human Rights Institutions |  |  |  |  |  |  |  |
| Politicians |  |  |  |  |  |  |  |
| Faith-based Organisations |  |  |  |  |  |  |  |
| Media |  |  |  |  |  |  |  |

1. Is there any water source in your area that you think can be developed to ensure availability of and access to water in your area? Yes No I don’t know
2. Are you aware that the United Nations has recognized water and sanitation as human right? Yes No

**THANK YOU FOR YOUR PARTICIPATION. PLEASE WRITE YOUR EMAIL OR PHONE NUMBER IF WE CAN CONTACT YOU FOR FURTHER CLARIFICATIONS**
